# Dissection of the Interplay between Class I PI3Ks and Rac Signaling in Phagocytic Functions

**DOI:** 10.1100/tsw.2010.178

**Published:** 2010-09-14

**Authors:** Carlotta Costa, Giulia Germena, Emilio Hirsch

**Affiliations:** Department of Genetics, Biology, and Biochemistry, Molecular Biotechnology Center, University of Turin, Italy

**Keywords:** PI3K, Rac, innate immunity, chemotaxis, phagocytosis, oxidative burst

## Abstract

Phagocytes, like neutrophils and macrophages, are specialized cells evolved to clear infectious pathogens. This function resides at the core of innate immunity and requires a series of concerted events that lead first to migration to the infected tissue and then to the killing of the invading pathogens. Molecular mechanisms underlying these processes are starting to emerge and point to the interplay between two families of crucial proteins: the PI3K lipid kinases and the Rac GTPases. This review focuses on how these two protein families contribute to migration, phagocytosis, and reactive oxygen species production, as well as their epistatic and feedback relations that finely tune these crucial aspects of the immune response.

## INTRODUCTION

Neutrophils and macrophages represent the first line of defense against microbial invasion and are key players in inflammatory processes. Sensing of inflammatory cues drives the recruitment of these cells to the site of inflammation through a directional movement called chemotaxis. Chemotaxis is a multistep process in which cell polarity, directional sensing, and cellular motility machineries are coordinated in order to generate efficient migration. After extravasation and recruitment to the inflammation site, the final goal of leukocytes is to eliminate invading micro-organisms through phagocytosis and destruction mediated by lytic enzymes and reactive oxygen species (ROS). In this review, we will focus on the role of Rac and PI3K signaling, and on the interplay between these two signaling proteins in phagocytic cells, particularly in neutrophils and macrophages, in migration, phagocytosis, and ROS production.

## MIGRATION

### PI3K and Leukocyte Migration

Phosphoinositide 3-kinases (PI3Ks) are enzymes that, by acting both as lipid and protein kinases, regulate several biological processes, including survival, proliferation, metabolism, and migration. PI3Ks are divided into three different classes (class I, II, and III) and class I members have been studied more

*These authors equally contributed to this work.
**Corresponding author.
©2010 with author.
Published by TheScientificWorld; www.thescientificworld.com extensively than the others. Class I PI3Ks are heterodimeric enzymes composed by a regulatory/adaptor subunit coupled to a catalytic subunit (called p110). Upon activation, all class I PI3Ks phosphorylate phosphatidylinositol(4,5)-bisphosphate ($PIP_2$), generating the lipid second messenger phosphatidylinositol(3,4,5)-trisphosphate ($PIP_3$). Depending on their activation mechanisms and their association with different regulatory subunits, these PI3Ks can be further divided into two subgroups, IA and IB. Class IA PI3Ks, comprising p110α-, β-, and δ-catalytic subunits, associate with a member of the p85 family adaptor proteins, and are activated both by receptor tyrosine kinases (RTKs) and G protein-coupled receptor (GPCRs). The unique member of class IB, p110γ, is activated exclusively by GPCRs and can associate with p84/p87 and p101 regulatory subunits. While α- and β-catalytic isoforms are ubiquitously expressed, γ and δ show a more restricted expression pattern, in particular in the hematopoietic lineage. Indeed, these two isoforms regulate different phagocytic functions and appear as crucial mediators of inflammatory reactions[1]. A large number of studies show that, in response to a wide range of stimuli, the loss of PI3Kγ leads to an impaired recruitment of neutrophils and macrophages to the site of inflammation[2,3,4,5,6]. In agreement, another report demonstrates that a PI3Kγ-selective inhibitor is three times more potent than LY294002 (a PI3K-pan inhibitor) in reducing neutrophil recruitment *in vivo*[7], thus suggesting that, in chemotaxis, a major role is specifically played by the p110γ isoform. However, a role for class IA in this process is still suggested by the finding that the tyrosine kinase inhibitor genistein inhibits $PIP_3$ generation in neutrophils[8]. Consistent with this view, *in vitro* experiments using a p110δ-selective inhibitor in human neutrophils indicate that p110δ has a role in controlling polarized morphology and chemotaxis. Authors propose that p110γ regulates the initial burst of $PIP_3$, while p110δ induces the amplification of $PIP_3$ production, leading to polarization and chemotaxis[9]. In further agreement, p110δ also affects chemotaxis of macrophages where it appears to be the main PI3K isoform recruited to tyrosine kinase receptors[10].

How p110δ as well as p110γ control cell polarization, directional sensing, or cellular motility is still debated. Chemoattractant stimulation of leukocytes induces a biphasic $PIP_3$ production; an initial transient and symmetric response around the cell membrane, followed by a second slower phase that amplifies differences in receptor occupancy, thereby achieving a highly polarized $PIP_3$ distribution[11,12,13,14]. Different studies suggest that PI3Kγ regulates cell polarity primarily by controlling polarization of $PIP_3$ and F-actin at the leading edge[13,15]. Nonetheless, PI3Kγ appears to control the number of cells moving in response to chemoattractants and is required for cell motility *per se*, but neither for speed nor directional sensing[16,17]. However, this mechanism is potentially operational only in cells migrating *in vitro*, in a bidimensional context. In agreement, a recent study in zebrafish demonstrates that PI3Kγ is required for neutrophil polarization and directional migration in a three-dimensional tissue environment[18], thus suggesting that PI3Kγ-dependent signaling events controlling cell motility and directional migration are tightly interconnected *in vivo*.

## Rac and Phagocyte Migration

Accumulation of $PIP_3$ at the leading edge of migrating phagocytes is thought to occur in response to local amplification events. Multiple evidences suggest that this is caused by positive feedback loops involving members of the Rho GTPase family that act as self-organizing and auto-amplifying signals[19]. Rho GTPases switch between an inactive state when associated with GDP and an active state when GTP bound. In resting conditions, the inactive Rho GTPase-GDP is cytosolic and generally associated to a GDI (GDP dissociation inhibitor). Upon stimulation, this complex is disassembled and the GTPase binds the membrane via its C-terminal prenylation sequence, allowing the exchange of GDP with GTP. In the GTP-bound state, these proteins can interact and activate different downstream targets, including kinases and regulatory proteins, ultimately controlling cytoskeletal remodeling[20]. Cycling of Rho GTPases is controlled by two classes of regulatory proteins: GEFs (guanine-nucleotide-exchange factors) that promote the exchange of GDP with GTP, and GAPs (GTPase-activating proteins) that stimulate the otherwise slow intrinsic GTPase activity[21].

The family of Rho GTPases is further divided into three groups: the Rho, Rac, and Cdc42 subfamilies. Since their discovery in the 1990s, these three subclasses have been found to play a pivotal role in signaling pathways that control morphogenesis and motility, mainly by regulating actin remodeling[20]. Nonetheless, Rac activity, but not that of Cdc42 or RhoA, is necessary and sufficient for chemoattractant-stimulated accumulation of actin polymers at the leading edge of migrating leukocytes[22]. Members of the Rac subfamily are thus emerging as critical regulators of phagocyte function. Rac regulates actin polymerization via different processes: (1) it increases availability of actin monomers for incorporation into actin filaments, (2) it favors free actin barbed-end formation through the removal of barbed-end capping proteins, and (3) it activates actin-nucleating proteins, including the Arp2/3 complex[20]. The Rac subfamily consists of three genes encoding Rac1, Rac2, and Rac3, respectively. These proteins are highly homologous, but display only slightly overlapping expression patterns. While Rac1 is ubiquitously expressed, Rac2 and Rac3 show a more restricted distribution, appearing enriched in the hematopoietic lineage and in the brain, respectively[23,24,25]. Rac1 plays a key role in the germ layer formation, as demonstrated by the embryonic lethal phenotype caused by its genetic ablation. Cells isolated from Rac1-deficient embryos indicate that Rac1 is involved in lamellipodia formation, cell adhesion, and migration *in vivo*[26]. Differently from Rac1, Rac2, and Rac3, knockout mice survive embryogenesis and show no obvious developmental defects[27,28]. How each Rac isoform contributes to phagocyte-specific functions comes from studies with conditional knockout mice. In macrophages, the loss of Rac1, the most abundant isoform in these cells, causes an elongated morphology and impaired spreading on adhesive surfaces, thus implying defective lamellipodia extension[29]. However, this mutation unexpectedly does not alter membrane ruffles formation and the speed of migration[29,30]. Similarly, macrophage migration in the absence of Rac2 alone or together with that of Rac1 shows limited defects, detectable in particular when cells are plated on a selected matrix such as laminin[31]. In contrast, Rac family members are strictly required for migration of neutrophils, where both Rac1 and Rac2 are expressed in equal amounts[32]. For example, Rac1 promotes gradient sensing[33], plays a role in tail retraction during cell movement[34,35], and controls, *in vivo*, neutrophil migration into the lung[36]. On the other hand, Rac2 regulates migration by controlling F-actin polymerization[27,32,33], thus demonstrating that, in neutrophil migration, Rac1 and Rac2 exert nonredundant roles[37]. Interestingly, this view is confirmed in human patients carrying a dominant-negative Rac2 mutant, where defects in neutrophil chemotaxis are observed[38,39].

## Crossroads of PI3K and Rac Pathways

Recent reports suggest that $PIP_3$ compartmentalization at the leading edge is caused by positive feedback loops that translate a shallow chemoattractant signal outside of the cell in a highly polarized cellular response, allowing cells to move toward the chemotactic gradient[19]. The hypothesis of the existence of a $PIP_3$ amplification mechanism, involving positive feedback loops (Fig. 1A), stems from a series of observations in cultured myeloid cells. For example, blockade of either Rho GTPases, PI3K, or actin polymerization significantly hampers $PIP_3$ accumulation at the leading edge[11,12,40]. In addition, delivery of exogenous $PIP_3$ is sufficient *per se* to trigger $PIP_3$ polarization, but this process is inhibited if either PI3K, actin, or Rho GTPases are blocked[11,12]. Finally, apparently contradicting reports in which PI3K activity is shown to function either upstream[41] or downstream of Rac activation[42,43] can be explained by the presence of a positive feedback loop between PI3K and Rac.

The mechanisms through which PI3K activates Rac are starting to emerge; for example, $PIP_3$ production at the leading edge promotes the localization of Rac activators containing the $PIP_3$-binding pleckstrin homology (PH) domain. In agreement, the mammalian Rac GEFs Tiam-1, Vav, and P-Rex1 all bind $PIP_3$ through their PH domains and regulate chemotaxis of various cell types[44,45,46]. Different members of the Rac GEF DOCK family are also found to regulate Rac activity in response to $PIP_3$. For example, during chemotaxis, they localize to the leading edge of chemotactic cells in a $PIP_3$-dependent manner. Interestingly, DOCK proteins do not possess a PH domain, but bind $PIP_3$ via the DOCK homology

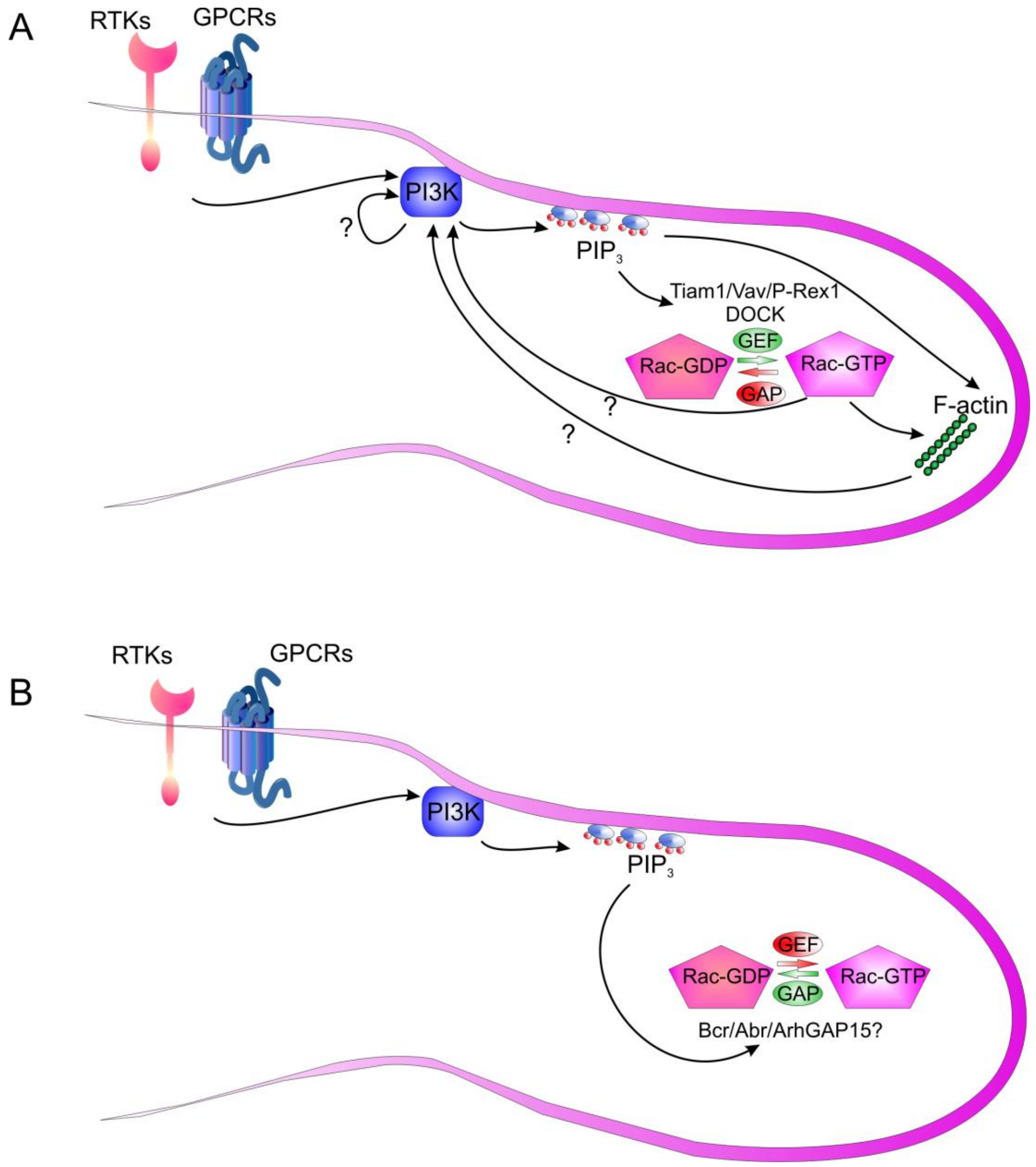

**FIGURE 1.** Schematic representation of the feedback loops involving PI3K and Rac at the leading edge of a migrating phagocyte. (A) Positive feedback loops involving PI3K, Rac, and actin cytoskeleton. (B) Potential negative feedback loops, controlled by PI3K and limiting Rac activity. GEF and GAP are represented in green when active and red when inactive, respectively.

region-1 domain (DHR-1)[47,48,49]. Moreover, a role in the feedback is likely played by selected Rac effectors of the WAVE/SCAR and WASP family of proteins, which regulate F-actin polymerization through their action on the Arp2/3 complex and can be regulated by $PIP_3$ docking[50].

Whereas $PIP_3$ activates Rac by promoting the organization of Rac-activating complexes, the molecular mechanism by which, in migrating phagocytes, Rac promotes $PIP_3$ accumulation is still obscure. Hints have recently emerged from studies in *Dictyostelium*, where Rac contributes to $PIP_3$ accumulation by promoting actin polymerization and the subsequent actin-mediated translocation of PI3K to the plasma membrane[51]. Nonetheless, in neutrophils, uniform activation of endogenous PI3K is apparently sufficient for $PIP_3$ polarization and effective cell migration. The observation that inhibitors of actin polymerization block these processes suggests that the mechanism described in *Dictyostelium* can also be similarly operational in neutrophils[11,12]. In contrast with this model, rapid activation of endogenous Rac independently of PI3K is sufficient to trigger effective actin polymerization, but fails to stimulate $PIP_3$ production or to induce cell polarization. It is thus possible that other mechanisms take place to reinforce PI3K activity independently of Rac[14]. While mathematical modeling of coexisting Rac-dependent and -independent PI3K activation mechanisms can accurately describe $PIP_3$ polarization[14], further studies are needed to define the molecular bases for this process.

The fact that $PIP_3$ production is initially symmetric and then amplified at the front suggests the presence of a negative feedback loop as part of a desensitization machinery that allows the cells to adapt to different stimulatory conditions[52]. Although molecules responsible for this negative feedback loop remain unidentified, it is possible to speculate that Rac GAPs have a role (Fig. 1B). Indeed, macrophages lacking both Abr and Bcr, two Rac GAPs, exhibit an atypical and elongated morphology, increased directional migration, and phagocytosis. Abr and Bcr contain a PH domain that could mediate their membrane translocation and activation[53]. Moreover, ArhGAP15, a PH-domain-containing Rac GAP, binds to and is activated by $PIP_3$ in migrating macrophages, suggesting that $PIP_3$ can control the GAP-dependent inactivation of Rac during chemotaxis[13]. It is thus possible that the interplay between $PIP_3$-dependent Rac GEFs and GAPs is necessary for the generation of pulsatile signaling required for fine tuning of cellular responses. How the identical $PIP_3$ recruitment controls Rac activation/deactivation cycles is currently unknown. However, differences in spatial and temporal patterns of membrane localization of distinct PH domain–containing proteins could be explained by a difference in the kinetics of their association and dissociation from the plasma membrane, which would be dictated by their affinity for $PIP_3$[19].

## PHAGOCYTOSIS AND ROS PRODUCTION

Phagocytosis is the mechanism used by immune system cells, such as macrophages, neutrophils, and dendritic cells, to ensure efficient clearing of pathogens and cell debris. Before phagocytosis, particles are coated on their surface by humoral immunity in a process called opsonization. Two distinct mechanisms of opsonization have been identified: particles coated with IgG bind the FcγR (Fragment, crystallizable γ Receptor), whereas particles coated with C3b fragments bind the complement receptor CR3 (Complement Receptor 3). While FcγR-dependent phagocytosis needs Rac and Cdc42 for membrane protrusions, it is commonly thought that CR3-dependent phagocytosis does not require membrane extension and depends on RhoA[54]. However, more recent evidences suggest that, in C3-dependent phagocytosis, Rac can also be implicated[55]. During phagocytosis three different steps are crucially required: (1) the phagocyte binds the invading particle (Fig. 2A), (2) the cell surrounds the particle with membrane protrusions called pseudopodia that together form the phagocytic cup (Fig. 2B), and (3) the particle is internalized in the proper phagosome (Fig. 2C) where it is degraded by lytic enzymes and by ROS production. In the killing of infective agents, ROS and their halogenated derivatives are key to the process because they act directly through their intrinsic chemical reactivity and indirectly through the activation of phagosomal proteases[56]. ROS generation is controlled by the NADPH oxidase complex that allows a one-electron reduction of $O_2$ to form superoxide anion ($O_2^-$). The phagocytic NADPH complex is composed by two transmembrane proteins ($p22^{phox}$ and $gp91^{phox}$, also called NOX2, which together form the cytochrome $b_{558}$) and a series of proteins that can shuttle from the cytosol to membranes, including $p40^{phox}$, $p47^{phox}$, $p67^{phox}$, and Rac. In resting conditions, the complex is inactive and the translocatable phox proteins are cytoplasmic; however, upon stimulation, phox proteins are relocated to the membrane and the assembly of the complex triggers NOX2 catalytic activity[57]. Membrane shuttling is tightly controlled by PI3K activity, which triggers Rac to assemble the active complex and provides lipid anchoring sites for phox proteins. In the following sections, we will thus focus on the involvement in ROS production and phagocytosis of class I PI3Ks and Rac, with particular attention on the collaborative network of interactions.

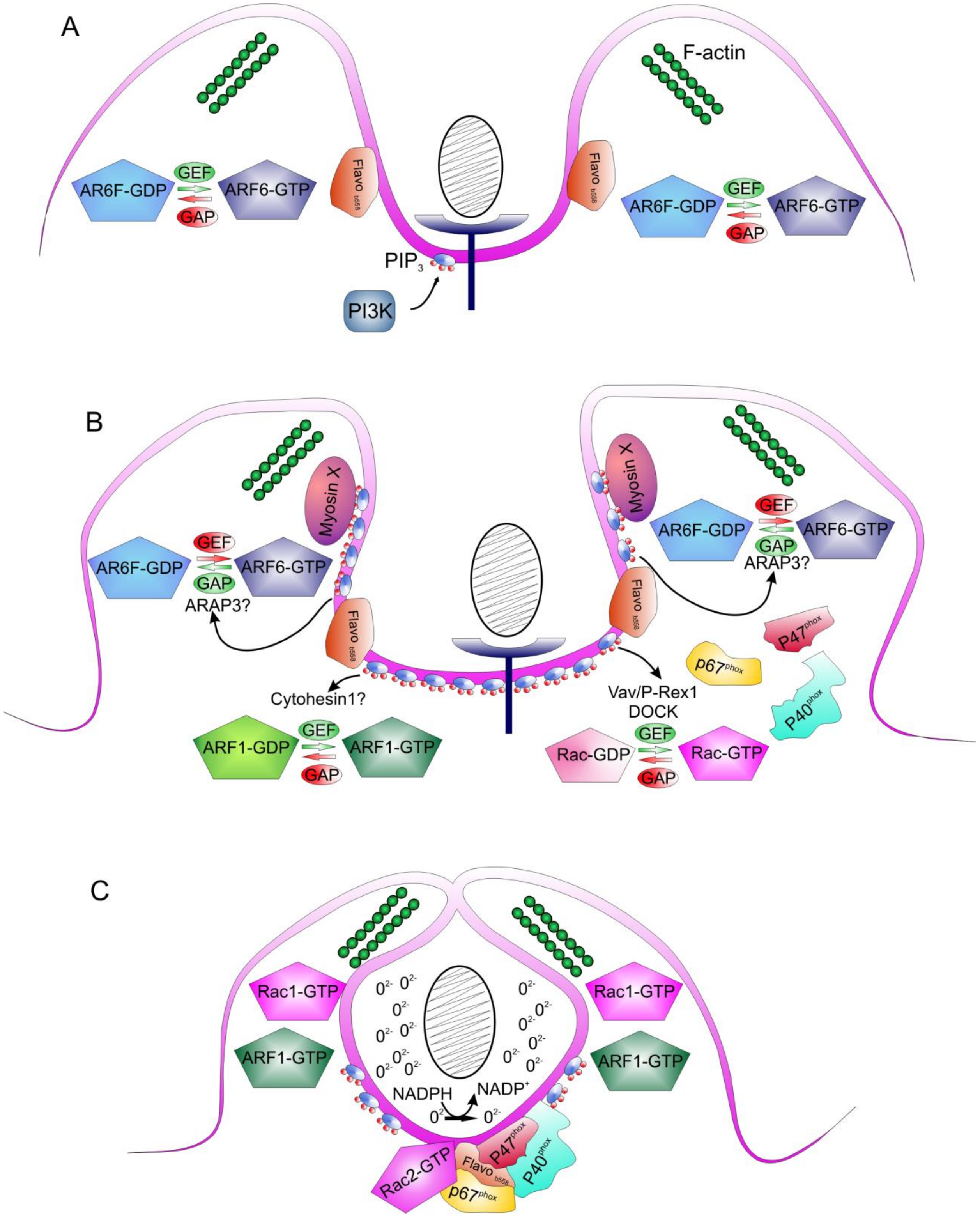

**FIGURE 2.** Schematic representation of the signaling events that, during phagocytosis and ROS production, involve PI3K and Rac activity. (A) Early activation events linked to receptor-mediated recognition of the particle to be ingested. (B) Signal transduction involving PI3K and Rac during pseudopodia extension. (C) $PIP_3$- and Rac-mediated events leading to the closure of the phagosomal cup and ROS production. GEF and GAP are represented in green when active and red when inactive, respectively.

## PI3K: Phagocytosis and ROS Production

Studies of the FcγR-mediated, multistep phagocytic process show that PI3Ks are required at selected stages. Consistently, $PIP_3$ transiently accumulates on the nascent phagosomal cup (the region of the cell close to the ingested particle) and disappears rapidly upon its closure[58,59]. While this PI3K activity has no role in the polymerization of F-actin at the phagocytic cup[60,61], it is involved in membrane extension around target particles as well as in the closure of pseudopodia into phagosomes[58,60]. In FcγR-mediated phagocytosis, class I PI3Ks also control exocytic membrane addition during phagocytic cup extension[62]. In these processes, PI3Ks might exert their classic action of controlling $PIP_3$-dependent docking and activation of different effector proteins. Consistent with this view, the PH domain–containing and $PIP_3$-binding protein, myosin X, is a key downstream molecule required for optimal extension of pseudopodia[63]. Moreover, delivery of new membranes to the growing pseudopodia is mediated by the PI3K-dependent control of ARF GTPases. ARF proteins can be positively and negatively regulated by PI3K; for example, in the forming phagosome, PI3K inhibits ARF6, but activates ARF1[64]. A plausible explanation for these observations resides in the possibility that $PIP_3$ production on phagocytic membranes stimulates GAPs deactivating ARF6 and GEFs activating ARF1[64]. Although at the moment there is no evidence about which GEFs and GAPs are involved in these processes, it is possible that PH domain–containing ARF regulators expressed in leukocytes might bind $PIP_3$ in consequence to PI3K activation. Potential examples are ARAP3, which is a ARF6 GAP[65,66], and cytohesin-1, which is a ARF1 GEF[67]. Whether specific class IA PI3K members selectively participate in these events is also presently unclear. Different reports point to either p110α or p110β, but results are controversial. For example, by microinjecting inhibitory antibodies, Leverrier et al. show that p110β,□ □ but not p110α, is required for FcγR-mediated phagocytosis in murine macrophages[68]. On the contrary, Lee et al. report that phagocytosis of IgG-opsonized zymosan is p110α dependent in human monocytic cells THP-1[69]. Nonetheless, species or cell-type differences might account for these divergent observations and further experiments are needed to address the issue. Interestingly, not all phagocytic processes require PI3Ks. Of note, while the role of PI3K signaling is clearly established in FcγR-mediated phagocytosis, recent evidences show that CR3-dependent phagocytosis is wortmannin insensitive, indicating that this event is mediated by PI3K-independent mechanisms[70].

Phagocytosis and oxygen species generation are coordinated and coincident events that allow efficient clearing of pathogens. Therefore, it is reasonable to hypothesize that these two processes may share molecular mechanisms coselected in evolution. In keeping with this hypothesis, PI3Ks control not only certain aspects of phagocytosis, but also of ROS production. Both macrophages and neutrophils treated with wortmannin show significantly reduced capacity to produce ROS following CR3-mediated phagocytosis. However, this effect appears to be independent of class I PI3Ks, as blockade of these isoforms does not inhibit ROS production triggered by CR3[70]. More critical in this process appears to be the class III PI3K Vps34, producing PI(3)P, but not $PIP_3$[70]. On the contrary, an involvement of class I PI3Ks is starting to emerge in FcγR-mediated oxidase activation. This is supported by the finding that inactivation of the $PIP_3$ 5-phosphatase SHIP-1, in mouse macrophages, blunts FcγR-evoked ROS production[71]. Interestingly, $p47^{phox}$ can bind the $PIP_3$ catabolite $PI(3,4)P_2$, allowing its translocation to intracellular membranes[72].The nature of the class I PI3K necessary for the initial $PIP_3$ production is, however, still unclear.

A better-defined function for class I PI3Ks in ROS production is revealed by the role of these enzymes in more specialized contexts where oxygen radicals are released not only in the phagosomes, but also in the extracellular milieu. Classical “phagocytic” receptors, such as CR3 and FcγR, act in synergy with cytokines and chemokines that potentiate ROS generation through GPCR-mediated signal transduction. Studies with wortmannin have been the first to reveal that PI3Ks are critically involved in the mechanisms leading to GPCR-mediated ROS release in neutrophils[73]. Further investigations in mice defined PI3Kγ as the major player in fMLP, C5a, and platelet-activating factor (PAF)–driven ROS production[2,3,4,74]. In contrast to these findings, the activation of the oxidase by fMLP in human neutrophils appears to rely on class IA rather than class IB PI3Ks[8,75,76]. An explanation for this apparent discrepancy comes from the use of isoform-selective PI3K inhibitors; while in mouse neutrophils ROS production is controlled by PI3Kγ alone, stimulation of primed human neutrophils causes a biphasic PI3K activation. The first phase is dependent on PI3Kγ, while the second phase, largely dependent on the first one, is mediated by PI3Kδ[77].

In summary, PI3K signaling shows a fine and granular distribution along the different lines that lead to ROS production from phagocytosis, suggesting that the same signaling module is shared by distinct, although coordinated, responses critical for phagocyte function.

## Rac: Phagocytosis and ROS Production

Phagocytosis requires concurrent actin assembly and pseudopod extension, two processes typically controlled by Rac GTPases. Consistently, concerted modulation of Rac1 and Rac2 is essential for the correct formation and closure of the phagocytic cup as well as the generation of ROS. Rac isoforms show nonoverlapping functions in controlling these processes with distinct roles in either neutrophils or macrophages. For example, in neutrophils, only Rac2 plays a role in both FcγR-dependent and complement-dependent phagocytosis[78,79,80]. In agreement, neutrophils from a human patient with a dominant-negative mutation of Rac2 show defective phagocytosis[38,39]. On the other hand, phagocytosis of macrophages relies on both Rac1 and Rac2, although each isoform can distinctly be activated by different phagocytic receptors. For example, Rac2-null macrophages display normal C3-dependent, but defective FcγR-mediated phagocytosis[30]. On the other hand, in the absence of both Rac1 and Rac2, C3-dependent phagocytosis is impaired[55], thus suggesting Rac1 as a major player in this specific process. In line with these functions, macrophages show that active Rac1 and Rac2 localize in phagosomal membranes, where they can control the closure of the phagocytic cup and regulate phagocyte actions aimed at pathogen elimination[81]. Interestingly, the importance of Rac activity in these processes is highlighted by the finding that multiple bacterial toxins regulate Rac function. These toxins potentially evolved to disguise phagocyte-mediated pathogen recognition and to hijack the phagocytic machinery to favor their survival. For example, *Salmonella typhimurium,* via its SopE protein, with Rac GEF activity, triggers Rac to promote its intracellular uptake necessary for proliferation in a protected environment[82]. Similarly, *Yersinia pseudotuberculosis* injects into host cells its effector protein YopE that, operating as a RacGAP, blocks its phagocytosis[83].

As mentioned above, Rac proteins also control pathogen clearing by regulating ROS production. Studies in genetically modified phagocytes show that Rac1 and Rac2 are not equally important in this process. While Rac1-null neutrophils display normal ROS production, Rac2-null neutrophils and macrophages exhibit a severely impaired superoxide generation[30,32,34,84]. In agreement, Rac2 can directly bind $p67^{phox}$ and induce its membrane translocation necessary for the activation of the NADPH oxidase complex[85]. This selective involvement of Rac2 is confirmed in human cells, as neutrophils from patients carrying a mutant Rac2 allele show severely defective superoxide production and impaired ability to clear bacterial infections[38,39].

## PI3K and Rac Meet to Trigger Phagocytosis and ROS Production

Results accumulated so far show that in phagocytosis and ROS production, Rac and PI3K regulate similar processes. It is thus reasonable to predict that, equally to what is seen in the migratory response, these two signaling proteins might be coupled by epistatic relations as well as feedback regulation mechanisms. Of note, different evidences show that PI3K functions upstream of Rac. For example, Rac2 activation is PI3K dependent in FcγR-mediated phagocytosis in macrophages[86]. Similarly, $PIP_3$ is a key component of chemoattractant receptor–stimulated pathways required for Rac2 activation leading to NADPH oxidase complex formation[87,88]. It is reasonable to predict that, in these events, PI3Ks activate Rac by regulating Rac GEFs. Good candidates for this function might be $PIP_3$-binding Rac GEFs like members of the Vav, DOCK, or P-Rex family. In agreement, macrophages lacking all Vav isoforms display a defect in complement-mediated phagocytosis[55] and NADPH activation[79]. Furthermore, in murine macrophages, knockdown of DOCK180 and its adaptor protein CrkII inhibits FcγR-dependent phagocytosis[89]. Finally, in neutrophils, P-Rex1 appears to be the crucial link between GPCR signaling, PI3K, Rac, and ROS production, as its binding to $PIP_3$ synergizes with the Gβγ subunits of heterotrimeric G proteins to activate the respiratory burst[41,90].

These evidences point out that different Rac GEFs are activated by PI3Ks in distinct cellular responses. How this selectivity is achieved is still mysterious and further studies are needed, for example, in order to detect the presence of preassembled complexes and/or subcellular $PIP_3$ production/demolition next to selected GEFs.

## PI3K AND RAC IN THE RESOLUTION OF INFLAMMATION

After having accomplished their defensive role, phagocytes are also critical for the resolution of the inflammatory reaction. For example, neutrophils leave the scene by apoptotic death and their debris is subsequently cleared by macrophages. Emerging evidence suggests that modulation of PI3K signaling might be involved in these biological responses. Activation of the PI3K/Akt pathway is well known to promote cell survival and signals stimulating these events can delay resolution of the inflammatory response. Among the large variety of PI3K-stimulating agents prolonging inflammation, it is interesting to mention cytokines like GM-CSF and TNF-α[91,92], growth factors like IGF-1[75], or infectious agents like respiratory syncitial virus[93]. A controversial role is instead played by the cAMP-activated signaling events. While cAMP can promote PI3K activation in cultured cells[94], other *in vivo* studies with peripheral blood-derived cells show that cAMP inhibits the PI3K pathway and thus promotes apoptosis[95]. Although a clear explanation for these potential discrepancies is not yet available, it is possible that subtle differences in cell type might influence the direction of the cAMP-mediated responses. Nonetheless, engagement of different PI3K isoforms could also be hypothesized. While this needs further investigation, a number of studies indicate PI3Kγ as a crucial isoform involved in controlling neutrophil survival. Consistent with this view, PI3Kγ-null neutrophils show increased levels of spontaneous and LPS-induced apoptosis[96], and PI3Kγ-null mice, characterized by an increased number of apoptotic infiltrating leukocytes in the brain, are protected in a model of autoimmune encephalomyelitis[97]. In further agreement, mice expressing a constitutive active isoform of PI3Kγ□ display a delay in the resolution of inflammation caused by an increased leukocyte survival[13].

Besides controlling apoptosis of neutrophils, PI3Ks are thought to control efferocytosis, the process of phagocyte-mediated clearance of apoptotic cells. In this context, only selected PI3K isoforms appear to play a role. Consistently, antibody-mediated inactivation of PI3Kβ causes a 70% reduction of phagocytosis of apoptotic cells[68]. Recent evidences also suggest that Rac positively regulates efferocytosis. Of note, glucocorticoid treatment causes increased macrophage engulfment of apoptotic cells and this process is related to enhanced Rac activity[98]. On the contrary, uPA (urokinase plasminogen activator) decreases Rac activity and consequently inhibits efferocytosis[99].

Taken together, these observations suggest that inhibition of PI3K signaling can potentially be a treatment of choice in disease conditions associated with abnormally prolonged inflammation like arthritis or chronic obstructive pulmonary disease. However, caution is needed because a potential side effect is the blockade of the efferocytotic process, which favors inflammation resolution. Further studies on the differential involvement of distinct PI3K isoforms in either neutrophil apoptosis or macrophage efferocytosis could help to address this concern by supporting the use of isoform-selective inhibitors.

## WHERE AND WHEN PI3K AND RAC MEET MATTERS

Through a positive feedback loop from $PIP_3$ to Rac and polymerized F-actin, and back to PI3K activity, Rac and PI3K work together optimally to promote migration, phagocytosis, and respiratory burst of phagocytes. Interestingly, precise tuning of these processes resides in the activation and control of positive feedback loops signaling to amplify minimal cue sensing locally. However, the complex relationship between PI3K and Rac is still far from being fully understood. Future studies are needed to better define how positive feedback loops are closed and how, for example, polymerized F-actin and Rac activate PI3K. Similarly, further investigations are required to understand how these positive feedback loops are dampened or terminated; for instance, better defining the function of those GAPs that can bind $PIP_3$ and inactivate Rac[13]. The understanding of these mechanisms will improve our abilities to manipulate phagocyte function and potentially help in the search for new therapies that effectively enhance their function in infectious diseases, but also, on the contrary, to dampen their activity in the course of pathologic inflammatory reactions.

## ACKNOWLEDGMENTS

We wish to thank Dr. Fulvio Morello for critically reading the manuscript. This work was supported by grants from Fondation Leducq, the European Union Sixth Framework Program EUGeneHeart, Telethon, Regione Piemonte, University of Torino, and AIRC.
